# Uterine Natural Killer Cell and Human Leukocyte Antigen-G1 and Human Leukocyte Antigen-G5 Expression in Vaginal Discharge of Threatened-Abortion Women: A Case-Control Study

**DOI:** 10.1155/2015/692198

**Published:** 2015-10-05

**Authors:** Saeideh Sadat Shobeiri, Zahra Rahmani, Hadi Hossein Nataj, Hossein Ranjbaran, Masoud Mohammadi, Saeid Abediankenari

**Affiliations:** ^1^Immunogenetics Research Center, Faculty of Medicine, Mazandaran University of Medical Sciences, Sari 48169 78741, Iran; ^2^Department of Obstetrics, Faculty of Medicine, Mazandaran University of Medical Sciences, Sari 48169 78741, Iran; ^3^Department of Immunology, Faculty of Medicine, Mazandaran University of Medical Sciences, Sari 48169 78741, Iran

## Abstract

The immunotolerant human leukocyte antigen-G (HLA-G) molecules have a major role in fetal-maternal tolerance during pregnancy. Interaction between these molecules and uterine natural killer (uNK) cells inhibitory receptors prevents NK cell invasion against fetus trophoblast cells. The aim of this study was to evaluate the percentages of uNK cells and HLA-G1 and HLA-G5 isoforms expression in vaginal discharge of threatened-abortion women in comparison with control. In a case-control study, we investigated 30 threatened-abortion women with bleeding or spotting less than 20 weeks of pregnancy as compared to 30 normal pregnant women. uNK cells percentage was assessed by flow cytometry. Furthermore, we evaluated HLA-G1 and HLA-G5 isoforms expression by Real-Time PCR in these groups. The results of this study showed that threatened-abortion women had increased uNK cells and decreased T cells percentage in vaginal discharge in comparison with normal pregnant women (*p* = 0.01, *p* = 0.003, resp.). In addition, HLA-G1 isoform had lower expression in threatened-abortion women in comparison with control group (*p* = 0.0001). The increase of uNK cells level with the decrease of HLA-G expression in vaginal discharge of threatened-abortion pregnant women is an indicator of mother's immune dysregulation. It is concluded that HLA-G expression level with uNK cells percentage can be determined as a diagnostic marker for threatened-abortion women.

## 1. Introduction

Spontaneous abortion is the most common complication during pregnancy [1, 2]. The prevalence of spontaneous abortion is about 12 percent which increases in next pregnancy [3–5]. Threatened-abortion occurs in 30–40 percent of pregnancies and it is identified as vaginal spotting or bleeding under 20 weeks of pregnancy [6]. The semiallogenic fetus antigens are in direct contact with the mother's immune system during pregnancy; therefore, immune suppression has a pivotal role in the maintenance of embryo [7].

HLA-G is a nonclassical HLA-Ib which has an important role in immune tolerance [8]. 50 alleles and 16 proteins are known for HLA-G [9]. This molecule has seven isoforms including HLA-G1, HLA-G2, HLA-G3, HLA-G4, HLA-G5, HLA-G6, and HLA-G7, where HLA-G1, HLA-G2, HLA-G3, and HLA-G4 isoforms are surface molecules and HLA-G5, HLA-G6, and HLA-G7 isoforms are soluble. In addition, soluble HLA-G1 originated from surface HLA-G1 by metalloproteinase protein activity [10, 11]. In physiologic conditions, expression of HLA-G is restricted to fetal trophoblasts at the maternal-fetal interface, cornea, thymic epithelium, pancreatic islets, nail matrix, and the erythroid and endothelial precursors [1, 12]. This molecule is also expressed in malignant transformation, autoimmune and inflammatory diseases, transplantation, and infectious diseases [1, 11]. It has been proven that HLA-G plays an important role in pregnancy. During pregnancy, these molecules can react with NK cells and T cell inhibitory receptors; thus, it can protect the fetal trophoblast cells from maternal uterine natural killer cells invasion [11].

Totally, human natural killer (NK) cells include 10–15 percent of circulating lymphocytes and are described phenotypically via their expression of CD56 and lack of CD3 expression [13]. Peripheral blood NK cells can be divided into two major subsets based on neural cell adhesion molecule CD56 (NCAM) expression including CD56^dim^ and CD56^bright^ NK cells. Cytolytic response is mostly restricted to the CD56^dim^ subset, while cytokine production is usually allocated to CD56^bright^ cells [13, 14]. Based on peripheral blood Nk cells level of CD56 expression and lack of CD3, NK cells can be divided into two major subsets. The two groups are defined as CD56^dim^ and CD56^bright^ NK cells. On the other hand, uNK cells are defined with high expression of CD56 (CD56^bright^) and lake of CD16 (CD-16^−^) and so they are phenotypically and functionally different from peripheral blood NK cells [15]. NK cells could play an important physiological role in preventing fetal trophoblast invasion and also these cells under circumstances could react with trophoblast cells, leading to abortion.

In this study, we evaluated HLA-G1 and HLA-G5 genes expression and uNK cells percentage in vaginal discharge of threatened-abortion women in comparison with normal pregnant women.

## 2. Material and Methods

In a case-control study, 30 threatened-abortion women and 30 normal pregnant women with the age range of the 19–42 years participated in the study (Table 1). The participants referred to obstetrics and Gynecology clinic in Mazandaran Province of Iran. The criteria for threatened-abortion women had been light spotting or vaginal bleeding before 20 weeks of pregnancy. On the other hand, diabetes, hyper- and hypothyroidism, immunological disorders, anatomic anomalies, and microbial infections, such as toxoplasmosis, rubella, and chromosomal abnormalities, were exclusion criteria of this study. These items were evaluated using patients' history, check-ups, and periodic examinations and collecting information through questionnaires approved by a gynecologist and an obstetrician. Participants were followed up until the end of their pregnancy.

### 2.1. Sample Preparation

A sample of cervicovaginal discharge was taken from each woman with sterile gynecological collector and was directly transferred to 1 mL RPMI-1640 medium (Sigma, USA). After 5 min centrifuge at 3000 RPM, the superior fluid was transferred to a microtube and kept at −70°C for the next ELISA analysis.

### 2.2. Isolation of Human Mononuclear Cells

The cellular suspension of vaginal discharge was added softly to Ficoll-Histopaque 1.077 (Biosera, UK) solution in a falcon tube and centrifuged at 400 g (2000 RPM) for 20 minutes. The loop containing mononuclear cells was collected and washed twice by RPMI-1640 medium.

### 2.3. Flow Cytometry

4 × 10^5^ vaginal discharge mononuclear cells were labeled with 5 *μ* of FITC-anti-CD3 monoclonal antibody and PE-anti-CD56 monoclonal antibody (eBioscience Inc., San Diego, CA, USA). These cells were evaluated by flow cytometry using Partec PAS III flow cytometer (Partec GmbH, Münster, Germany). The gate was set around the lymphocytes on forward scatter and side scatter dot plot to disclose the other cells of the following analysis. Interference of FL1 and FL2 signals was adjusted by compensation using FITC and PE antibodies. 5 × 10^4^ lymphocytes were evaluated for each test.

### 2.4. Quantitative Real-Time Polymerase Chain Reaction (qPCR)

Total RNA was isolated from vaginal discharge using CinnaPure RNA extraction kit (SinaClon BioScience Co., Tehran, Iran), according to the manufacturer's procedure. Total RNA was reversely transcribed using the AccuPower CycleScript RT PreMix (dN 6) kit (Bioneer Inc., Seoul, South Korea). qPCR was performed using specific primers for HLA-G1, HLA-G5 isoforms and elongation factor 1 (reference gene) genes (Table 2) with SYBR Green qPCR Master Mix.

Quantitative PCR reactions were done in a 20 *μ*L volume including 1x SYBR Green PCR master mix (Bioneer Inc., Seoul, South Korea), 1 *μ*L of forward and reverse primers, and 2 *μ*L of cDNA following the manufacturer's instructions. After a primary 10 min at 95°C as activation step, 40 cycles comprising of denaturation at 95°C, 30 s, annealing at 59°C, 30 s, and extension at 72°C, 45 s were performed by BioRad iQ5 Multicolor Real-Time PCR (Bio-Rad Laboratories, Hercules, CA, USA) detection system. Gene expression was analyzed by comparing with the reference gene in each PCR run.

### 2.5. Enzyme-Linked Immunosorbent Assay (ELISA)

IL-10 level in superior fluid of vaginal discharge cellular suspension was measured by an ELISA Ready-SET-Go kit (eBioscience, USA) according to the manufacturer's procedure.

Briefly, 100 *μ*L capture antibody in coating buffer (48 *μ*L capture antibody (250x) in 12 mL coating buffer per plate) per well of a 96-well plate (Corning Costar flat-bottom plates, eBioscience, USA) was incubated overnight at 4°C. Each well was then blocked with 200 *μ*L of 1x Assay Diluent (1 part 5x concentrated Assay Diluent with 4 parts DI water) for 1 hour at room temperature, followed by overnight incubation with 100 *μ*L of sample at 4°C. Then, each well was incubated with detection antibody diluted in 1x Assay Diluent (48 *μ*L detection antibody in 12 mL 1x Assay Diluent) for 1 hour at room temperature. Each incubation step was followed by 3 washes with wash buffer (PBS including 0.05% Tween 20). 100 *μ*L of avidin-HRP diluted in 1x Assay Diluent was added to each well. Following 7 washes with wash buffer, 100 *μ*L of substrate solution was added to each well and the plate was incubated for 15 min at room temperature. Reactions were stopped by addition of 50 *μ*L stop solution (1 M H_3_ PO_4_: phosphoric acid). Plate was read at 450 nm with ELISA reader.

### 2.6. Statistical Analysis

For statistical analysis, we used student's *t*-test and Mann-Whitney test. The *p* values were characterized in all cases and the *p* < 0.05 was considered to be statistically significant.

## 3. Results

Totally, sixty pregnant women participated in this case-control study. Out of the 30 threatened-abortion women, 26 pregnant women were followed up during their pregnancy. 53.84 percent (14/26) of the threatened-abortion women pregnancies resulted in abortion.

### 3.1. uNK, NKT, and T Cells in Vaginal Discharge Mononuclear Cells

**Figure 1:**
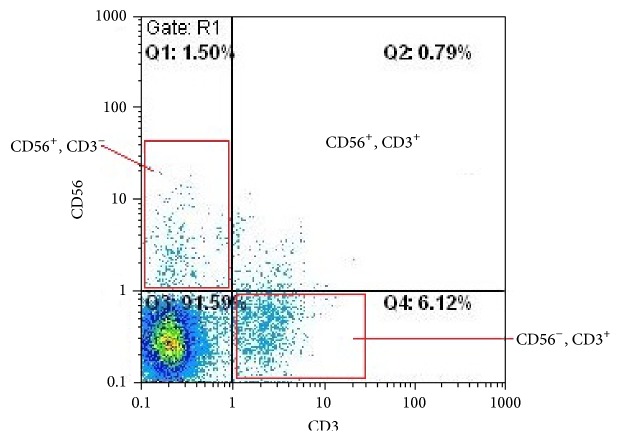
Dot plot analysis of vaginal discharge mononuclear cells, FL1 (CD3) and FL2 (CD56). CD56^+^3^−^ NK cells are found in the upper-left panel. CD56^−^3^+^ T cells are found in the lower-right panel. The mononuclear cells were isolated from vaginal discharge by Ficoll-Histopaque (1.077) and stained with FITC-anti-CD3 and PE-anti-CD56 monoclonal antibody. Gate was set around the lymphocytes. Fifty thousand lymphocytes were analyzed by flow cytometry.

The population of NK, NKT, and T cells in vaginal discharge mononuclear cells is presented in Figure 1.

**Figure 2:**
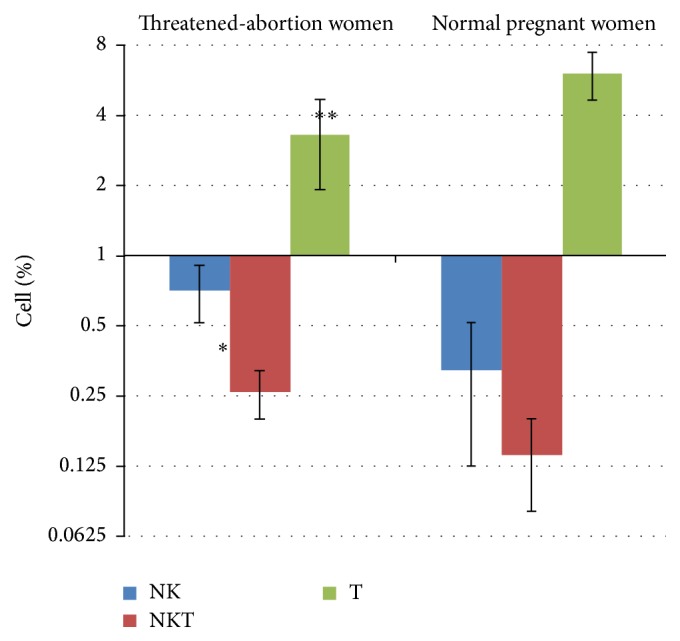
Percentage of NK, NKT, and T cells in the threatened-abortion women as compared to the control group (mean ± SEM).

The result showed that uNK cells level significantly increased in vaginal discharge of threatened-abortion women in comparison with normal pregnant women (*p* = 0.01). Moreover, the level of T cells in threatened-abortion women was significantly lower than control group (*p* = 0.003). There was no significant difference in NKT cells percentage between the two groups (*p* = 0.85) (Figure 2).

### 3.2. HLA-G Isoforms Expression in Vaginal Discharge

**Figure 3:**
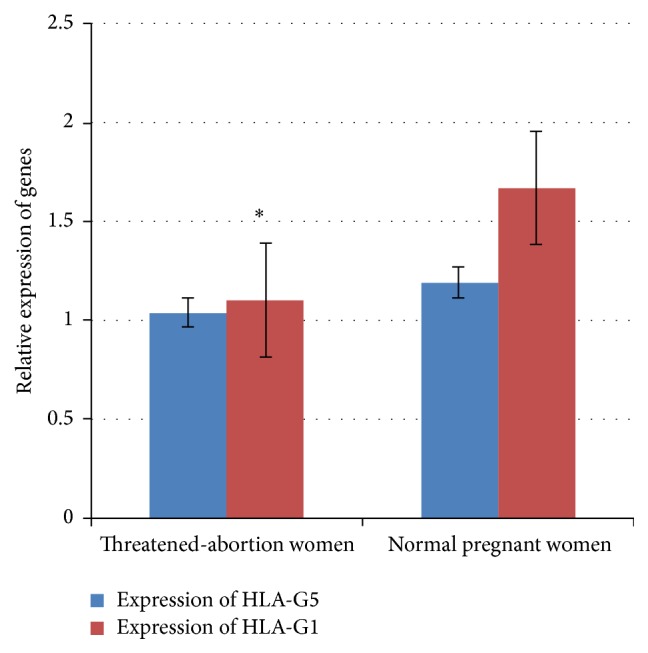
The comparison of relative expression of HLA-G1 and HLA-G5 isoforms in threatened-abortion women and control group (mean ± SEM).

HLA-G1 expression was significantly lower in the vaginal discharge of threatened-abortion women in comparison with normal pregnant women (*p* = 0.0001). However, threatened-abortion women had a decreased level of HLA-G5 in comparison with control group, but this difference was not significant (*p* = 0.12, Figure 3).

### 3.3. Interleukin-10 Secretion in Vaginal Discharge

The concentration of IL-10 was determined in the vaginal discharge via ELISA as an indicator of cytokine production. This cytokine production level in threatened-abortion women was lower compared with the normal pregnant control group (3.91 ± 1.46 versus 4.76 ± 2.5). But the difference was not significant (*p* = 0.3).

## 4. Discussion

At first, we carried out an evaluation on NK cell count and expression of HLA-G isoforms expression (pan HLA-G, HLA-G1, HLA-G5) in the vaginal discharge of the threatened-abortion women in comparison with control group, which was determined before 20 weeks of pregnancy. Previously, it was observed that cell-bearing HLA-G acquires tolerogenic potential to downregulate various immune reactions [12]. Our results showed that HLA-G1 and HLA-G5 isoforms decreased in the abortion-threatened women (Figure 3). It means that these molecules are transiently upregulated during pregnancy. Formerly, we showed that HLA-G isoforms levels are different among individuals [11]. In this research, we found that the HLA-G1 expression in vaginal discharge was higher than HLA-G5 in healthy pregnant women and HLA-G1 levels in vaginal discharge of the threatened-abortion women are significantly higher in comparison with HLA-G5 expression in healthy pregnant women. Therefore, it is recommended that the evaluation of HLA-G1 expression in vaginal discharge can be used as a normal pregnancy marker. Probably, expression of HLA-G1 isoform is controlled by key vital factors for preservation of fetus. In addition, HLA-G1 may be a major isoform in the tolerance of mother's immune system. Furthermore, it is possible that the low expression of HLA-G1 contributed to allogenic NK cell proliferative response in semiallogenic fetus. On the other hand, HLA-G1 and HLA-G5 have an important role in the upregulation of T regulatory cells in the pregnant women [16]. Also, our results showed that the high expression of HLA-G resulted in an excess of IL-10 in the vaginal discharge. Thus, HLA-G1 is an inhibitory constituent and may exert a key suppressive effect on T cell proliferation response to semiallogenic fetus with the cooperation of HLA-G bearing cells inhibitory cytokine secretion.

In this study, it was observed that the percentage of natural killer cells increased in the vaginal discharge of the threatened-abortion women in comparison with normal pregnant women which could be an indicator of fetus loss. In addition, low expression of HLA-G1 immunotolerance molecule is a risk factor for spontaneous abortion in comparison with control group. Furthermore, our data showed that HLA-G1 expression was significantly lower in abortion-threatened women in comparison with the control group. However, HLA-G5 expression level was not significantly different between the two groups, which propose that HLA-G5 has inconsiderable characteristic in the mentioned population.

Several studies have shown a significant correlation between peripheral blood natural killer cell and HLA-G in abortion-threatened women. In addition, it is reported that NK cell level can be a predictor of abortion in pregnant women [17, 18]. Furthermore, there is a strong association between HLA-G and NK cell level with spontaneous abortion [19]. Thus, NK cell activity may be an important marker for pregnant women [20]. It is suggested that predictive value of HLA-G or NK cell is superior to other established prognostic factors for fetal loss [21]. Carosella et al. reported that HLA-G can be the most relevant marker for abortion screening due to T cell response [22]. On the other hand, HLA-G identified as a marker for tolerance and its plasma level was associated with better survival in pregnant women [23, 24]. Our results show a significant correlation between vaginal discharge IL-10 level with high HLA-G and low NK cell count. These findings show a positive correlation between HLA-G1 and HLA-G5 expression with IL-10 level and a negative correlation between NK cell counts with these cytokines.

## 5. Conclusions

According to the results, HLA-G molecules contribute to the regulation and control of the immune response.

In conclusion, the results of our study showed that vaginal discharge, NK cell percent, and HLA-G1 and HLA-G5 expression were correlated with abortion in comparison with control. Therefore, uterine NK and HLA-G1 and HLA-G5 have an important key role in fetal surveillance during pregnancy.

## Figures and Tables

**Table 1:** Demographic characteristics and clinical parameters of threatened-abortion women and normal pregnant women.

Variable	Normal pregnant women	Threatened-abortion women
	*N* = 30	*N* = 30
	Mean ± SD	Mean ± SD
Age (year)	29.7 ± 5.75	28.83 ± 5.58
Weight (kg)	65.9 ± 6.7	67.0 ± 9.5
Gestational age (week)	9.5 ± 4.24	8.97 ± 4.2
Hemoglobin (g/dL)^1^	12.11 ± 0.77	12.46 ± 0.83
Hematocrit (percent)	34.7 ± 1.26	35.8 ± 1.81
WBC (K/*μ*L)^2^	8.25 ± 1.9	9.25 ± 1.81
RBC (mil/*μ*L)^3^	4.2 ± 0.35	4.35 ± 0.39
Recurrent abortion (*n*)	—	0–2
Familial disorder	Negative	Negative
VDRL	Negative	Negative
HBs Ag	Negative	Negative
Immunosuppressive drugs intake	No	No
Smoking	No	No

^1^gram per deciliter.

^2^Thousands per cubic milliliter.

^3^Millions per cubic millimeter.

**Table 2:** Primer sequence of HLA-G1, -G5 isoforms and elongation factor 1.

Gene	Primer	Sequence	Product length (bp)	Accession number
EF-1	Forward	CTGAACCATCCAGGCCAAAT	59	XM_011535514
	Reverse	GCCGTGTGGCAATCCAAT		

HLA-G1	Forward	CTGGTTGTCCTTGCAGCTGTAG	80	XM_011547651
	Reverse	CCTTCCTTACCTGAGCTCTTCTTTCT		

HLA-G5	Forward	CGGAGTATTGGGAAGAGGAGA	384	NM_002127.5
	Reverse	TGGTACCCGCGCGCTGCAG		
